# Substantial deficiency of free sialic acid in muscles of patients with GNE myopathy and in a mouse model

**DOI:** 10.1371/journal.pone.0173261

**Published:** 2017-03-07

**Authors:** Yiumo Michael Chan, Paul Lee, Steve Jungles, Gabrielle Morris, Jaclyn Cadaoas, Alison Skrinar, Michel Vellard, Emil Kakkis

**Affiliations:** Ultragenyx Pharmaceutical Inc., Novato, California, United States of America; University of Valencia, SPAIN

## Abstract

GNE myopathy (GNEM), also known as hereditary inclusion body myopathy (HIBM), is a late- onset, progressive myopathy caused by mutations in the *GNE* gene encoding the enzyme responsible for the first regulated step in the biosynthesis of sialic acid (SA). The disease is characterized by distal muscle weakness in both the lower and upper extremities, with the quadriceps muscle relatively spared until the late stages of disease. To explore the role of SA synthesis in the disease, we conducted a comprehensive and systematic analysis of both free and total SA levels in a large cohort of GNEM patients and a mouse model. A sensitive LC/MS/MS assay was developed to quantify SA in serum and muscle homogenates. Mean serum free SA level was 0.166 μg/mL in patients and 18% lower (p<0.001) than that of age-matched control samples (0.203 μg/mL). In biopsies obtained from patients, mean free SA levels of different muscles ranged from 0.046–0.075 μg/μmol Cr and were markedly lower by 72–85% (p<0.001) than free SA from normal controls. Free SA was shown to constitute a small fraction (3–7%) of the total SA pool in muscle tissue. Differences in mean total SA levels in muscle from patients compared with normal controls were less distinct and more variable between different muscles, suggesting a small subset of sialylation targets could be responsible for the pathogenesis of GNEM. Normal quadriceps had significantly lower levels of free SA (reduced by 39%) and total SA (reduced by 53%) compared to normal gastrocnemius. A lower SA requirement for quadriceps may be linked to the reported quadriceps sparing in GNEM. Analysis of SA levels in *Gne*^*M743T/M743T*^ mutant mice corroborated the human study results. These results show that serum and muscle free SA is severely reduced in GNEM, which is consistent with the biochemical defect in SA synthesis associated with *GNE* mutations. These results therefore support the approach of reversing SA depletion as a potential treatment for GNEM patients.

## Introduction

GNE myopathy (GNEM), also known as hereditary inclusion body myopathy (HIBM), distal myopathy with rimmed vacuoles (DMRV) or Nonaka disease, is a rare, late-onset, progressive muscle wasting disease [1]. The disease is characterized by symptom onset in humans typically between the ages of 20 and 40 years with weakness in distal muscles and subsequently extended to proximal muscles of the lower and upper extremities with relative sparing of the quadriceps [2]. GNEM is also allelic to distal myopathy with rimmed vacuoles (DMRV) and Nonaka disease sharing striking clinical and pathological similarity. Rimmed vacuoles are a distinct pathological feature of the disease and intracellular protein aggregates, such as β-amyloid, phosphorylated tau, TAR DNA-binding protein 43kD, α-synuclein, were often detected in the rimmed vacuoles by immunohistochemistry. The genetic basis of GNEM is caused by mutations in the UDP-N-acetylglucosamine 2-epimerase/N-acetylmannosamine kinase (*GNE*) gene [3–5]. *GNE* encodes a bi-functional enzyme (GNE/MNK) with epimerase and kinase activity and is responsible for the first two rate-limiting steps in the biosynthesis of N-acetylneuraminic acid (Neu5Ac or NANA), the most common form of sialic acid (SA) [6–8]. GNEM is more prevalent in certain ethnic groups, for example, in the Persian Jewish population that predominantly carries a common founder M743T mutation in the *GNE* gene [9–11].

Sialic acid is an essential sugar for modification of proteins and lipids required to maintain normal cellular functions. The critical role of GNE/MNK in cell survival and development was confirmed in the *Gne* knockout mouse which resulted in embryonic lethality [12]. Given the putative function of the *GNE* gene, the disease mechanism is thought to be primarily associated with deficiency in SA production in skeletal muscle [13–16]. One hypothesis is that the disease arises from the hyposialylation of a yet-to-be-identified protein(s) critical for normal muscle function [17]. Alternatively it has also been proposed that *GNE* plays an independent role from SA synthesis, including interaction with α-actinins [18, 19], apoptosis via mitochondrial dysfunction [20] and modulating expression levels of ST3Gal5 and ST8Sia1 sialyltransferase [21]. Today, it remains unclear how mutations in the *GNE* gene precisely affect the biochemistry of SA and pathology in GNEM [22, 23]. This controversy can be partly attributed to the difficulty in accurate measurement and differentiation of different SA pools in tissues. In addition to being chemically attached to the terminal ends of the glycans on glycoproteins or lipids (bound SA), SA can also exist as a smaller soluble pool in tissues (free SA). Thus, total SA was defined as the sum of free and bound SA. Currently, the impact of GNE/MNK defect on the levels of the smaller but critical pool of free SA in GNEM has not been thoroughly explored. The assessment of SA levels is further hampered by the sensitivity of SA assays being used in most studies. Previous studies in GNEM patients and in animal models have mostly focused on total SA as a measure of cellular sialylation but with conflicting results. Noguchi *et al* have reported that there was no difference in total SA levels in serum samples between patients and normal controls, whereas in skeletal muscle, a 25% reduction of total SA was observed in a small number of patients using the thiobarbituric acid method [13]. In contrast, no reduction in cellular sialylation was detected in B lymphoblastoid cell lines derived from GNEM patients using colorimetric assays and lectin analysis, despite the fact that the GNE/MNK enzyme showed reduced activity [24, 25].

Recently, several GNEM animal models have been developed to understand the underlying biochemical and cellular defects in the disease as well as to explore potential therapies for GNEM. A transgenic mouse model (DMRV-hIBM) overexpressing human *GNE* D176V mutation in null GNE background recapitulated many of the pathological changes in skeletal muscles reminiscent of GNEM in terms of rimmed vacuole and inclusion body formation [26]. In this mouse model, total SA levels in serum and muscle membrane-bound fractions were decreased compared with control littermates. Both reversal and prophylactic treatments with SA or SA metabolites in the DMRV-hIBM mice led to increased serum and muscle total SA levels as well as ameliorated or prevented development of myopathic phenotypes [27–29]. In a different mouse knockin model (*Gne*^*M743T/M743T*^ mouse) which harbors the human equivalence of homoallelic M743T mutation, oral treatment of SA in adult mutant mice for 12 weeks improved muscle sialylation as demonstrated by lectin histochemistry [30, 31]. Altogether, these animal studies indicated that SA deficiency as measured by total SA in muscle is a component of the disease and thus provided the rationale for developing oral SA treatment for GNEM patients.

An unusual clinical feature of GNEM is the initial sparing of the quadriceps, although this muscle group can be partially impaired at a later stage. Muscle wasting and weakness in GNEM shows a striking degree of specificity for different muscles. For example, tibialis anterior, iliopsoas and biceps femoris are more severely and preferentially affected than other muscle groups [2, 32]. So far, there is no good explanation for this differential involvement pattern. The levels of free and total SA in different human muscles have not been extensively studied and it is not known whether there are variations in SA levels and thus sialylation requirement for different muscles. Additional work is therefore required to assess the levels of free and total SA in different muscles in GNEM to better understand the disease pathology and quadriceps sparing.

In this study, we conducted a comprehensive and systematic assessment of free and total SA levels in serum and biopsies of different muscles collected from a large number of clinically and genetically defined GNEM patients (n = 47). The main objective was to evaluate SA deficiency in GNEM and to assess free and total SA in different muscles in order to understand the impact of SA levels on the pathogenesis of GNEM. A sensitive and validated LC/MS/MS assay was developed for this purpose to quantify SA in serum and muscle homogenates. Levels of both free and total SA were also determined in the *Gne*^*M743T/M743T*^ mouse model. The results obtained from this study will allow better assessment of SA deficiency in patients with GNEM and provide useful insights into the underlying disease mechanism that guides the design and development of therapy for GNEM.

## Materials and methods

### Acquisition of GNEM patient serum samples and muscle biopsies

Serum samples and muscle biopsies were collected from 47 GNEM patients who enrolled in a phase 2 randomized, double-blind, placebo-controlled, parallel group study to evaluate the dose and pharmacodynamic efficacy of aceneuramic acid extended release (AceER) tablets (ClinicalTrials.gov: NCT01517880). The protocol was approved by the New England Institutional Review Board (reference #12–133) on 18-May-2012. The protocol and consent documents were also approved by each clinical site’s IRB and each of the consents included a checkbox for whether or not the subject’s samples were allowed to be used for research purposes. Samples were collected before treatment began to assess baseline SA levels. Muscle specimens from both the lower and upper extremities were collected by needle biopsy and were flash frozen in liquid nitrogen. Lower extremity samples were collected from the quadriceps (n = 37) and gastrocnemius (n = 10). Upper extremity samples were collected from the deltoid (n = 40) and biceps (n = 7).

### Acquisition of control serum and muscle samples

All control serum and muscle samples were procured from tissue banks in compliance with all applicable federal and state laws and regulations and ethical guidelines. Control serum samples from age-matched donors (16 males and 31 females, range 18–78 years old) were acquired from BioChemed Services (Winchester, VA, http://www.biochemed.com). Donors were without a history of renal, endocrine, hepatic, autoimmune and neoplastic disease. Serum was allowed to clot spontaneously prior to separating the serum from the cellular components by high speed centrifugation and maintained as individual pools. Control human quadriceps muscle samples (vastus lateralis) from autopsy donors without a history of muscle disease (8 males and 4 females, range 34–66 years old) were obtained from BioreclamationIVT (Westbury, NY, http://www.bioreclamationivt.com) and Integrated Laboratory Services Biotech (Charlestown, MD, http://www.ils-inc.com). Selected samples were examined by H&E staining to ensure muscle quality. Control human deltoid muscle samples from autopsy donors without a history of muscle disease (2 males and 3 females, range 60–69 years old) were obtained from BioreclamationIVT. Control human gastrocnemius muscle samples from autopsy donors or lower leg amputatees without a history of muscle disease (5 males and 4 females, range 48–86 years old) were obtained from BioreclamationIVT and BioOptions (Brea, CA, http://biooptions.com). All muscle samples were collected within 24h post-mortem and snap-frozen in liquid nitrogen.

### GNEM mouse model

The *Gne*^*M743T/M743T*^ mice were kindly provided by Dr. Daniel Darvish and Dr. Yadira Valles-Ayoub at HIBM Research Group (Reseda, CA). The mouse model was engineered with a knockin M743T (ATG to ACG) mutation in the exon 12 of murine *Gne* gene equivalent to the human M743T mutation. The mouse model has been described previously as *Gne*^*M712T/M712T*^ mice [30, 31]. The adaptation to the new nomenclature of *Gne*^*M743T/M743T*^ was due to recent discovery of an additional N-terminal sequence of 31 amino acids [11]. These mice, when maintained in B6 background, have been shown to develop severe kidney abnormalities and die at an early postnatal stage. However, the *Gne*^*M743T/M743T*^ mice are viable when maintained on a mixed FVB;B6 background [33].). The genotype of all mice used in the study was confirmed by PCR of tail genomic DNA followed by restriction digest with *NlaIII* (Idexx Radil, Columbia, MO). This assay can distinguish wild type, heterozygous and homozygous animals. It is based on a one base pair change in the mutant allele that eliminates a *NlaIII* restriction site found in the wild-type allele. PCR primer set consisted of forward primer: 5’-AGC ACT TCC TGG AGT TTG ATG-3’ and reverse primer: 5’-ATT TGC CTT CGC AGA AAC ACT GA-3’. Control littermates of wild-type and heterozygous mice were obtained from the same colony. All mice were housed in BioTox Sciences (BTS), (San Diego, CA 92121) according to animal care guidelines of the facility. All animal studies and procedures were approved by the Biotox Sciences Institutional Animal Care and Use Committee (IACUC) and conducted employing sound scientific practices and in accordance with the written study plan and BTS Sciences Standard Operating Procedures. Animal welfare was in compliance with the U.S. Department of Agriculture’s (USDA) Animal Welfare Act (9 CFR Parts 1, 2, and 3), the Guide for the Care and Use of Laboratory Animals. The animal facilities at BioTox Sciences are accredited by Association for Assessment and Accreditation of Laboratory Animal Care International (AAALAC) International. The animals were monitored daily by observing for indications of pain and distress as characterized by behavioral changes including increased or decreased activity, CNS effects, huddling, loss of appetite, lethargy, ruffled fur, marked body weight changes and vocalization. The decision to euthanize clinically ill or moribund animals due to indications of pain and distress was the responsibility of the Study Director, in collaboration with the Attending Veterinarian and the Sponsor’s Study Monitor. Euthanasia on study animals was performed as scheduled or if required due to morbidity via inhalation of isoflurane followed by exsanguination. Euthanasia was conducted in accordance with accepted American Veterinary Medical Association (AVMA) guidelines.

### Creatine and protein assays

Creatine levels in muscle specimens were assayed using EnzyChrom Creatine Assay Kit (ECRT-100; BioAssay Systems) according to the manufacturer’s protocol. Prior to assay on human samples, the Creatine Assay Kit was qualified using creatine (Sigma) as a reference for the calibration curve and quality control. Creatine concentrations were read at OD_570nm_ using an Emax Microplate Reader with SoftMax Pro Software. The range of the assay was 100–1000μM. Creatine/wet tissue weight ratio (Cr/weight ratio) for each muscle specimen was expressed in μmol/g using the formula based on the tissue extraction volume of 0.3mL (0.0003L): creatine conc. ([μmol/L] x 0.0003L)/ tissue wet (g). Protein concentrations in mouse muscles were assayed by micro-Lowry method using bovine serum albumin (Sigma) as reference standard. Protein concentrations were read at OD_570nm_ using Emax Microplate. The range of the assay was 1.5-20mg/mL.

### Exclusion of poor quality muscle specimens based on creatine content

Creatine/weight ratio (μmol creatine/g wet tissue) was used as a criterion for excluding muscle specimens contaminated with non-muscle tissue. To determine the normal range for different muscles, creatine/weight ratio was first assessed in control samples. Both control quadriceps and deltoid samples had a mean creatine/weight ratio of 19.18–21.19 μmol/g whereas control gastrocnemius samples had a slightly lower mean creatine/weight ratio of 13.04 μmol/g (Table 1A). The range of creatine/weight ratio in the normal muscle controls was in agreement with the reported values for human muscles in the literature [34]. A value equivalent to the mean normal creatine/weight ratio minus 1SD was then arbitrarily selected as a cutoff for each muscle (1SD below mean in Table 1A). If a muscle specimen had a creatine/weight ratio below this cutoff value, the specimen was considered as poor quality from non-muscle contamination and thus excluded from muscle SA analysis. Most of the normal muscle controls met the criteria of minimal creatine content with the exception of 2 of 12 quadriceps controls and 2 of 9 gastrocnemius controls. In contrast, a substantial number of GNEM biopsies, especially for gastrocnemius (70%) and deltoid (55%) did not achieve the inclusion criterion (Table 1B and 1C).

**Table 1:** Creatine/weight ratio of human control muscles and exclusion criteria for muscle specimen. Unit was expressed in μmol of creatine/g of wet tissue weight. The cutoff value was defined as mean creatine/ratio minus 1SD (Table 1A). Total number of control and GNEM biopsy specimens available and number of specimens that met the cutoff criteria were listed in the Tables 1B and 1C.

**Table 1A**	**control quads (n = 12)**	**control GS (n = 9)**	**control deltoid (n = 5)**
mean	19.18	13.04	21.19
1 SD below mean (cutoff)	13.43	8.44	17.4
**Table 1B**	**control quads**	**control GS**	**control deltoid**
total # specimens	12	9	5
# specimens met cutoff criteria	10 (83%)	7 (78%)	5 (100%)
**Table 1C**	**GNEM quads**	**GNEM GS**	**GNEM deltoid**
total # specimens	37	10	40
# specimens met cutoff criteria	29 (78%)	3 (30%)	18 (45%)

### Development and validation of LC/MS/MS assay for SA measurement

The SA assay was developed by and performed at Intertek Pharmaceutical Services (El Dorado Hills, CA). Free SA in human serum samples was assayed as follows. NANA was extracted from 50 μL of human serum by protein precipitation using methanol: acetonitrile (3:1) followed by centrifugation at 3400 rpm for 15 mins. ^13^C_3_-NANA was spiked into the samples as an internal standard at 10 μg/mL. After filtration, evaporation to dryness, and reconstitution in 5mM NH_4_OAc in water and the extracts were analyzed by LC-API/MS/MS. Separation and elution of free SA was achieved by reverse-phase liquid chromatography (Shimadzu LC-20ADXR HPLC) with Hypercarb™ 100 x 3 mm (5 μm) column (Thermo Scientific). Solvent for mobile phase A was 0.1% formic acid in water and for mobile phase B, solvent was acetonitrile with 0.1% formic acid. The initial flow rate was 0.8 mL/min and adjusted to 1.2 mL/min at t = 2.23 min and returned to 0.8 mL/min at t = 5.31 min. The total run time was 6 min with gradient in mobile phase B from 8% (t = 0.01 to 2.00 min), 12% (t = 2.00 to 2.20 min), 90% (t = 2.21 to 3.50 min), and 8% (t = 3.51 to 5.31 min). The analytical column temperature was kept at 30°C and the injection volume was 10 μL. Detection of free SA was determined by AP Sciex API-4000 triple quadrupole mass spectrometer (Applied BioSystems) under electrospray ionization operated in negative ion mode. Spray voltage was -3800 V and the in-source CID energy was set at -23 V and capillary temperature was 550°C. Run times were approximately 6 minutes. NANA has m/z transition of 308.2 → 87.1 (parent mass → fragment mass). Dialyzed 5% BSA was used as a surrogate matrix for the calibration curve. The Lower Limit of Quantitation (LLOQ) for free SA in serum was 0.04 μg/mL (Table 2). The range of reliable response was 0.04–20.0 μg/mL. This bioanalytical procedure was based on the results of a full validation study in human serum, using dialyzed 5% BSA as a surrogate matrix.

**Table 2:** Range of free and total NANA in the LC/MS/MS assay for human serum, muscle homogenate and urine samples.

	free NANA (μg/mL)	total NANA (μg/mL)
**serum**	0.04–20	NA
**muscle**	0.02–20	0.3–100
**urine**	1–200	5–400

Methodology for analysis of free SA in muscle was identical to the protocol for free SA in serum with the exception that NANA was extracted from 20 μL of human muscle homogenate. Dialyzed 2% BSA was used as a surrogate matrix for the calibration curve and low QC; all other QC levels were prepared by spiking control porcine muscle homogenate with NANA (1000 μg/mL) to obtain concentrations of 40 μg/mL (Acceptable Quality Limit, AQL), and 15 μg/mL (high QC) and 6 μg/mL (mid QC). After preparation, QC samples were stored at −70°C until analysis. The Lower Limit of Quantitation (LLOQ) for free SA in muscle was 0.02 μg/mL (Table 2). The calibration curve range was 0.02–20.0 μg/mL. Sialic acid in muscles that is bound to glycoproteins and lipids can be released for quantification with acid hydrolysis. Total SA was therefore the sum of free SA and bound SA released by acid hydrolysis. To assay total SA, 20 μL of human muscle homogenate was treated with H_2_SO_4_ at 80°C in the presence of 40 μg/mL ^13^C_3_-NANA internal standard. After centrifugation and dilution into 5mM NH_4_OAc in water, the extracts were analyzed by LC-API/MS/MS as described above. Aqueous salt/organic compound solution was used as a surrogate matrix for the calibration curve and low QC; all other QC levels were prepared in porcine muscle homogenate. The Lower Limit of Quantitation (LLOQ) for total SA in muscle was 0.3 μg/mL (Table 2). The calibration curve range was 0.03–100.0 μg/mL. The calculated concentrations of acceptable analytical QCs must be within 15% of the nominal concentration; at least two-thirds of all analytical QCs shall meet the given criteria, with at least 50% of the QC samples at each level meeting criteria. Absolute concentrations of human muscle SA (μg/mL) in each sample were corrected for creatine and expressed as normalized concentration in μg/μmol of creatine using the formula: absolute NANA conc. (μg/mL)/creatine (μmol/L x [1L/1000mL]). Absolute concentrations of mouse muscle SA (μg/mL) in each sample were corrected for protein and expressed as normalized concentration in μg/mg of protein using the formula: absolute NANA concentration (μg/mL)/protein concentration (mg/mL).

### Extraction of muscle tissues

For human muscle homogenate preparation, the biopsy sample was thawed in an ice bath and then weighed. The sample was kept cool during the entire procedure. Muscle biopsies were homogenized in 300 μL of chilled Tris Buffer/Protease Inhibitor (TB/PI) Solution: Dissolved one Roche cOmplete Mini Protease Inhibitor Cocktail Tablet in 10 mL of 10mM Tris, pH 7.5. Homogenization was performed using the Omni TH homogenizer with a 5 mm probe. Final muscle homogenates were sub-aliquotted and flash-frozen on dry ice, or immediately transferred to -70°C freezer for storage until assayed for SA and creatine. Mouse muscle homogenates were prepared following the same human muscle preparation protocol with the exception that 200mM NH4OAc, pH 4.4 was used instead of Tris buffer.

### Statistical analysis

Descriptive statistics including means, medians, standard deviations and counts were used in the study. For data comparison between GNEM and controls, two-tailed paired t-test was used. If the data were not normally distributed, Wilcoxon’s rank sum test was employed. SigmaPlot, version 12.5, was used for all analyses. A p-value of < 0.05 was considered statistically significant. Error bars in the graphs show standard error (SE).

## Results

### Quantification of sialic acid by LC/MS/MS

Accurate quantification of both free and total SA in biological samples is essential to understand the biochemistry of SA in GNEM and to account for subtle changes in SA levels. Previous of studies have mostly focused on total SA due to abundance and ease of detection. To precisely quantify free SA in biological samples, we have developed a sensitive and specific LC/MS/MS assay for measuring NANA in serum and muscle tissue that was based in part from a previous method [35]. The LLOQ for free SA in human serum and muscle homogenate in our LC/MS/MS-based assay was 0.04 μg/mL and 0.02 μg/mL, respectively (Table 2). The sensitivity of the LC/MS/MS-based assay was significantly improved compared with other commonly used colorimetric assays based on thiobarbituric acid which generally has a detection range of 1–2 μg/mL.

### Serum free SA is reduced in GNEM patients and *Gne*^*M743T/M743T*^ mice

**Fig 1:**
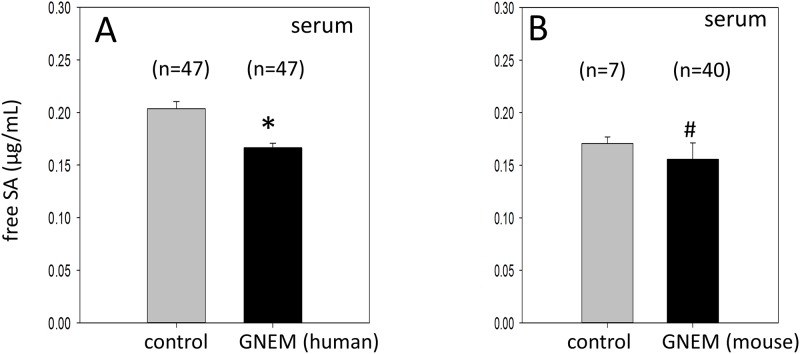
Serum SA levels in GNEM patients (A) and *Gne*^*M743T/M743T*^ mice (B) compared to normal controls. Values are expressed as means ± SE. * -indicates a statistical significant difference with a p<0.001. ^#^—indicates a statistical significant difference with a p<0.009.

The levels of free SA in serum collected from GNEM patients and normal controls were determined by LC/MS/MS. The mean serum free SA level in GNEM patients was 0.166 μg/mL which was 18% lower than the level in age-matched normal controls (0.203 μg/ml, p <0.001) (Fig 1A). Similarly, the mean serum free SA level in our *Gne*^*M743T/M743T*^ mice was also significantly lower compared with aged-matched controls (0.156 μg/mL for mutant mice vs 0.171 μg/mL for controls; p = 0.009) (Fig 1B).

### Censoring of muscle SA data in GNEM patients

One of the challenges for accurate quantification of muscle SA levels was the quality and quantity of the muscle biopsies obtained from patients. In this study, the majority of the lower and upper extremity muscle biopsies were collected from quadriceps and deltoid, respectively. Quadriceps biopsies were likely to contain sufficient muscle tissue but might not be the ideal muscle for analysis due to the quadriceps sparing characteristic. On the other hand, the small number of gastrocnemius and biceps biopsies collected from patients was more likely to be contaminated with non-muscle tissues compared to healthy muscles because of the pathological changes.

**Fig 2:**
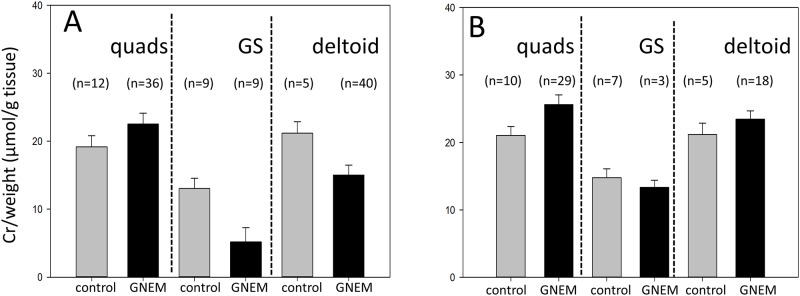
Creatine/weight ratio of normal muscle controls and GNEM patient biopsies according to muscles. Quads = quadriceps, GS = gastrocnemius. **A)** All available samples. **B)** Samples remained after excluding specimens with low creatine/weight ratio as defined in Table 1.

To address the potential variation in muscle biopsy quality, we used creatine/weight ratio (μmol creatine/g wet tissue) as a criterion to exclude muscle specimens contaminated with non-muscle tissues which would have an impact on muscle SA quantification and thus the interpretation of the data. Given that more than 95% of creatine is found in skeletal muscles [36], a low creatine/weight ratio would indicate the presence of substantial non-muscle tissues in the specimens. The criteria for excluding inappropriate muscle specimens are summarized in Table 1. The mean creatine/weight ratios for different muscles for both normal controls and GNEM patients before and after exclusion are shown in Fig 2A and 2B, respectively. After applying the exclusion criteria, the creatine/weight ratio between GNEM patients and normal controls for the same muscle were within comparable ranges.

### Free SA represents a small fraction of total SA in muscle

**Fig 3:**
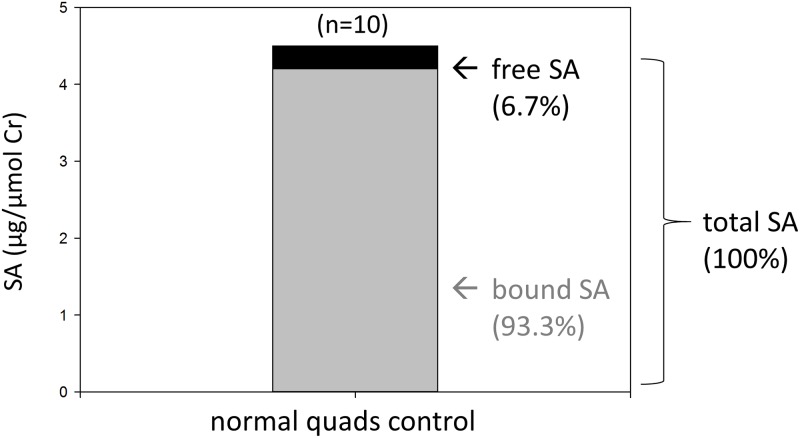
Free, bound and total SA in normal quadriceps controls. SA values were corrected for creatine (μg SA/μmol creatine).

We assessed both free and total SA levels in human skeletal muscles using the LC-MS/MS assay. Previous studies have focused primarily on total SA levels, perhaps due to less sensitive assays. Sialic acid concentration (μg/mL) in muscle homogenates was normalized using creatine and expressed as μg of SA/μmol of Cr. In quadriceps controls that met the creatine/weight ratio criteria, the mean normalized free SA level in muscle biopsies was 0.299 μg/μmol Cr and the mean normalized total SA level was 4.474 μg/μmol Cr (Fig 3). Thus the free SA content constituted only a small fraction (6.7%) of the total SA in quadriceps. Similarly, free SA also represented a small percentage of total SA in gastrocnemius (5.1%) and deltoid controls (3.5%).

### Muscle free SA is significantly reduced in GNEM patients

**Fig 4:**
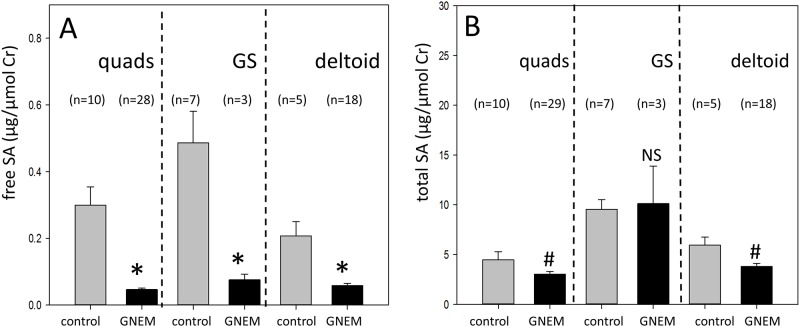
Free and total SA levels of normal muscle controls and GNEM patient biopsies according to muscles. **A)** Free SA data after excluding specimens with low creatine/weight ratio as defined in Table 1. * -indicates a statistical significant difference with a p<0.001 for quadriceps (quads), p = 0.025 for gastrocnemius (GS), p = 0.002 for deltoid. **B)** Total SA data after excluding specimens with low creatine/weight ratio as defined in Table 1. ^#^ -indicates a statistical significant difference with a p = 0.02 for quads, p = 0.008 for deltoid. NS denotes not statistically significant. Free and total SA values were corrected for creatine (μg SA/μmol creatine).

Free SA levels in valid (after removal of low creatine samples) GNEM muscle biopsies across muscles (quadriceps, gastrocnemius, and deltoid) were significantly reduced compared with normal controls (Fig 4A). Mean free SA in quadriceps was 85% lower in GNEM patients compared with normal controls (0.046 vs 0.299 μg/μmol Cr). Gastrocnemius (0.075 vs 0.487 μμg/μmol Cr) and deltoid (0.058 vs 0.207 μg/μmol Cr) muscles of GNEM patients also showed similar reductions of 85% and 72%, respectively compared with normal controls. Interestingly, free SA level in normal quadriceps controls was 39% lower than that of normal gastrocnemius controls (p-value = 0.09).

### Muscle total SA is moderately decreased in GNEM patients

The exclusion of inappropriate specimens had a significant impact on the total SA values of GNEM gastrocnemius and deltoid (data not shown). This is consistent with our concern that biopsies obtained from affected gastrocnemius and deltoid were more likely to be contaminated with non-muscle tissues compared to quadriceps biopsies due to fibrous and fatty tissue replacement. Total muscle SA levels were lower in quadriceps and deltoid samples from GNEM patients that met the creatine/weight ratio criteria (Fig 4B). In quadriceps of GNEM patients, the total SA level was 3.032 μg/μmol Cr compared with the normal controls level of 4.474 μμg/μmol Cr (32% reduction). In deltoid of GNEM patients, the total SA level was 3.795 μg/μmol Cr compared with the normal control level of 5.941 μg/μmol Cr (36% reduction). These decreases in total SA were less than those observed for muscle free SA but were statistically significant compared to control muscles. In contrast, no significant difference was observed in total SA levels for gastrocnemius between GNEM patients (10.117 μg/μmol Cr) and normal controls (9.547 μg/μmol Cr). Similar to the pattern observed for free SA, normal quadriceps controls also had significantly lower total SA level compared with gastrocnemius controls (53% reduction, p = 0.001).

### Free and total SA in muscles of GNEM mice showed similar extent of decrease to GNEM patients

**Fig 5:**
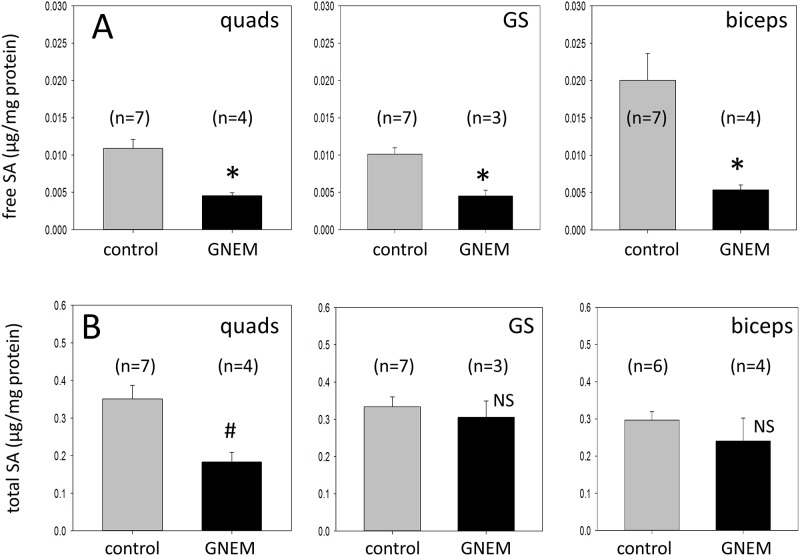
Free and total SA levels of wild-type controls and *Gne*^*M743T/M743T*^ mice according to muscles. **A)** Free SA data in both littermate controls and *Gne*^*M743T/M743T*^ mice. * -indicates a statistical significant difference with a p = 0.006 for quadriceps (quads), p = 0.005 for gastrocnemius (GS), p = 0.006 for biceps. **B)** Total SA data in both littermate controls and *Gne*^*M743T/M743T*^ mice. ^#^ -indicates a statistical significant difference with a p = 0.01 for quadriceps (quads). NS denotes not statistically significant. Free and total SA values were corrected for protein (μg SA/mg protein).

Both free and total SA levels were assessed in quadriceps, gastrocnemius and biceps muscles in *Gne*^*M743T/M743T*^ mice >1 year old. The levels of free SA in all three different muscles were significantly reduced in the HIBM mice compared to age-matched control littermates (Fig 5A). Free SA levels were lower by 73% in biceps (0.0054 vs 0.020 μg/mg protein) of *Gne*^*M743T/M743T*^ mice compared with normal controls. Free SA levels in the quadriceps (0.0046 vs 0.011 μg/mg protein) and gastrocnemius (0.0045 vs 0.010 μg/mg protein) of the mutant mice also showed similar reductions of 58% and 55%, respectively, compared with control littermates. Our results demonstrated that both GNEM patients and our *Gne*^*M743T/M743T*^ mice displayed a similar and significant reduction in free SA in all muscles examined (Figs 4A and 5A).

Total SA reduction was more variable across different muscles from *Gne*^*M743T/M743T*^ mice but showed a similar trend to GNEM patients (Fig 5B). A statistically significant reduction of 48% in total SA was observed in the quadriceps (0.183 vs 0.351 μg/mg protein) compared to control littermates but not in gastrocnemius (0.305 vs 0.333 μg/mg protein) or biceps (0.240 vs 0.297 μg/mg protein). The free and total SA data in both GNEM patients and *Gne*^*M743T/M743T*^ mice are summarized in Table 3.

**Table 3:** Summary of free and total SA results in serum and muscle for human and mouse data. Sialic acid values were shown as mean normalized values with SE. Change of SA in GNEM patients or *Gne*^*M743T/M743T*^ mice from corresponding controls were shown as percentage change. Statistical not significant change was represented as p = NS.

	Human Data			Mouse Data		
	Human Controls	GNEM Patients	Change in GNEM patients from controls	Littermate Controls	HIBM Mice	Change in GNEM mice from controls
**serum free SA**	0.203±0.007 μg/mL (n = 47)	0.166±0.004 μg/mL (n = 47)	-18% (p<0.001)	0.171±0.006 μg/mL (n = 7)	0.156±0.016 μg/mL (n = 40)	-9% (p = 0.009)
**quads free SA**	0.299±0.050 μg/μmol Cr (n = 10)	0.046±0.004 μg/μmol Cr (n = 28)	-85% (p<0.001)	0.011±0.001 μg/mg (n = 7)	0.0046±0.0004 μg/mg (n = 4)	-58% (p = 0.006)
**GS free SA**	0.487±0.094 μg/μmol Cr (n = 7)	0.075±0.020 μg/μmol Cr (n = 3)	-85% (p = 0.025)	0.01±0.001 μg/mg (n = 7)	0.0045±0.0008 μg/mg (n = 3)	-55% (p = 0.005)
**deltoid free SA**	0.207±0.043 μg/μmol Cr (n = 5)	0.058±0.007 μg/μmol Cr (n = 18)	-72% (p = 0.002)	NA	NA	NA
**biceps free SA**	NA	NA	NA	0.02±0.004 μg/mg (n = 7)	0.0054±0.0007 μg/mg (n = 4)	-73% (p = 0.006)
**quads total SA**	4.474±0.802 μg/μmol Cr (n = 10)	3.032±0.266 μg/μmol Cr (n = 29)	-32% (p = 0.025)	0.351±0.036 μg/mg (n = 7)	0.183±0.026 μg/mg (n = 4)	-48% (p = 0.01)
**GS total SA**	9.547±0.958 μg/μmol Cr (n = 7)	10.117±3.766 μg/μmol Cr (n = 3)	6% (p = NS)	0.333±0.027 μg/mg (n = 7)	0.305±0.044 μg/mg (n = 3)	-8% (p = NS)
**deltoid total SA**	5.941±0.809 μug/μmol Cr (n = 5)	3.795±0.320 μg/μmol Cr (n = 18)	-36% (p = 0.008)	NA	NA	NA
**biceps total SA**	NA	NA	NA	0.297±0.023 μg/mg (n = 6)	0.240±0.062 μg/mg (n = 4)	-19% (p = NS)

## Discussion

In this study, we evaluated and quantified both free and total SA levels in serum and skeletal muscles of clinically and genetically-defined GNEM patients to gain a better understanding of the relationship between SA deficiency and GNEM. To the best of our knowledge, this is the first comprehensive report to systematically investigate both free and total SA levels in such a large cohort. Our results demonstrated free SA levels in muscle and serum were consistently and significantly lower in GNEM patients than in control specimens. Total SA levels in muscles of GNEM patients were also reduced compared with normal controls but the reduction was less than that observed in free SA and was not consistent across all muscles examined. Thus our results clearly show for the first time a major deficiency in muscle free SA in GNEM in addition to moderate and variable reductions in muscle total SA. Sialic acid levels were also determined in the *Gne*^*M743T/M743T*^ mouse model for the first time and the mouse data paralleled those in GNEM patients. In particular, mean free SA also demonstrated a drastic reduction in all three muscles of the mutant mice examined compared with control littermates. Previous studies did not assess muscle SA levels in this mouse model due to the early post-natal lethality. When maintained on a mixed FVB;B6 background, our *Gne*^*M743T/M743T*^ mice survived for more than one year.

The corroboration of the free SA results between the GNEM patients and our *Gne*^*M743T/M743T*^ mice suggests a strong and consistent relationship between SA biosynthesis and SA levels consistent with a biochemically meaningful deficiency. Both GNEM mice and humans showed the same significant reductions in muscle free SA (3–5 fold) compared with normal control levels despite the fact that free SA constitutes only approximately 3–7% of the total SA pool in muscle. The biological significant of free SA deficiency is not clear and our results demonstrated that it is a common mechanism of GNEM, consistent with the function of *GNE* in SA synthesis. Free SA acid must undergo activation to the CMP-SA nucleotide carrier sugar intermediate in the nucleus in order to be incorporated into either proteins or lipids by sialyltransferases in the Golgi. Although CMP-SA is thought to be the cellular pool of SA for glycoconjugate incorporation, one could speculate that the establishment of an adequate level of intracellular pool of free SA is essential for subsequent CMP-SA production and sialylation in muscles. It will be of interest to determine whether CMP-SA levels are also deficient in GNEM. In addition, the generation of SA and CMP-SA is an energetically costly process since it involves the utilization of multiple high energy phosphate bonds from glucose to CMP-SA. The intracellular level of SA is also tightly regulated in humans via negative feedback inhibition of GNE/MNK enzyme by CMP-SA [37]. The production of free SA is therefore a highly regulated cellular process in order to meet cellular demands on an as-needed basis and its levels might be particularly sensitive to disruption in SA biosynthesis. Thus maintenance of an adequate free SA pool could be critical for muscle function.

In contrast, reductions in muscle total SA were more moderate and variable depending on the muscles examined. The levels of reduction reported in this study for GNEM patients was in general agreement with the 25% reduction of total SA in muscle previously observed in a small number of GNEM patients [13]. When interpreting our total SA results, the impact of non-muscle contamination in the patient biopsies must be carefully taken into account. In this study, an exclusion criteria based on creatine/tissue weight ratio was designed to assess muscle quality. This approach had a relatively large impact on total SA values rather than free SA values of GNEM patient biopsies, especially those obtained from gastrocnemius and deltoid biopsies, which were more likely to be contaminated with non-muscle tissues compared with quadriceps. However, it remained difficult to completely eliminate the contribution of total SA from minor contaminants in the biopsies. Taken together, our results indicated that changes in cellular sialylation in GNEM muscles could be more subtle than previously thought. The depletion of both free and total muscle SA in GNEM could reduce levels below a critical general threshold to maintain normal sialylation in muscle. However, we cannot rule out the possibility that a small number of specific sialylation targets are involved in GNEM. Currently, little is known about the specific targets for sialylation that might be responsible for the pathogenesis of GNEM. A number of proteins, for example, Neural Cell Adhesion Molecule (NCAM) [38, 39], alpha-dystroglycan [40, 41] and neprilysin [28, 42] have been proposed as candidates, but there is lack of direct evidence to demonstrate their role in GNEM. Recently, it has been shown the ratio of desialylated Thomsen-Friedenreich (T) antigen and sialylated form, ST-antigen was altered in plasma of GNEM patients [43]. Although the specific glycoproteins involved remained to be identified, the study indicated that hyposialylation of core 1 O-linked glycans could play a predominant role in GNEM.

An interesting finding in this study was that SA levels in human quadriceps were less those in gastrocnemius whereas we did not see such difference in mice. In particular, total SA level in human quadriceps was significantly lower than gastrocnemius. These observations suggested that the overall SA requirement for human quadriceps may be lower compared with gastrocnemius and that this may be a unique feature to biped humans. The lower demand for SA by the human quadriceps might effectively protect the tissue from SA deficiency in GNEM and therefore account for the quadriceps-sparing characteristic that is observed in humans but not the *Gne*^*M743T/M743T*^ mouse model. Likewise, a higher SA demand by the gastrocnemius could result in more severe and rapid progression of the disease in the distal muscles. Based on this argument, there might be a threshold level for SA required for normal function in humans that could vary in different muscles or developmental stages. Supporting this hypothesis, a previous study has shown that free and total SA excreted in urine of normal individuals declined with age and particularly at puberty, consistent with the adult onset of GNEM [35]. Children might therefore be protected then from the impact of the GNE mutation on their muscles by higher levels of GNE/MNK induction until late childhood and puberty occurs. In addition, lectin staining of quadriceps from normal elderly people demonstrated a decrease in SA-specific SNA staining compared with younger people [44]. In agreement with the threshold effect, it has been shown that small changes in serum total SA levels resulting from SA treatment in the DMRV-hIBM mice led to considerable improvement in pathology and muscle function [27].

Although it is not clear why SA levels in human quadriceps were less than that of in gastrocnemius, we noticed that the creatine content was higher in quadriceps compared to gastrocnemius. Unlike the type I slow-twitch fibers, type II fast-twitch fibers utilize the glycolytic pathway for energy and also rely heavily on the creatine-phosphocreatine for rapid generation of ATP. It has been shown the basal phosphocreatine levels were higher in type II than in type I fibers [45]. These observations indicate a potential difference in metabolic requirements which could impact the overall SA threshold levels in different muscles. One could speculate that different metabolic requirements could contribute to the difference in SA synthesis and levels observed for quadriceps vs gastrocnemius. As a result, it is possible that different muscles could display different sialylation patterns and that some specific glycococonjugates when hyposialylated could lead to different functional consequences.

In summary, we have developed a sensitive LC/MS/MS method to quantify SA levels in serum and muscle tissues. This report provided direct evidence of substantial free SA deficiency in GNEM patients and our *Gne*^*M743T/M743T*^ mice. Although we cannot exclude the possibility that the GNE gene is involved in other cellular mechanisms, our results are consistent with a substantial biochemical defect in SA synthesis that underlies GNEM and supports the approach of developing SA substrate replacement therapy to replenish the free SA pool for treating GNEM patients.
